# Mechanisms and Opportunities for Rational In Silico Design of Enzymes to Degrade Per- and Polyfluoroalkyl Substances (PFAS)

**DOI:** 10.1021/acs.jcim.3c01303

**Published:** 2023-11-20

**Authors:** Melissa Marciesky, Diana S. Aga, Ian M. Bradley, Nirupam Aich, Carla Ng

**Affiliations:** † Department of Chemical and Petroleum Engineering, University of Pittsburgh, Pittsburgh, Pennsylvania 15213, United States; ‡ Department of Chemistry, State University of New York at Buffalo, Buffalo, New York 14260, United States; § Department of Civil, Structural, and Environmental Engineering, State University of New York at Buffalo, Buffalo, New York 14228, United States; ∥ Department of Civil and Environmental Engineering, University of Nebraska—Lincoln, Lincoln, Nebraska 68588-0531, United States; ⊥ Department of Civil and Environmental Engineering, University of Pittsburgh, Pittsburgh, Pennsylvania 15261, United States; ▽ Research and Education in Energy, Environmental and Water (RENEW) Institute, University at Buffalo, The State University of New York, Buffalo, New York 14260, United States

**Keywords:** PFAS, enzyme design, bioremediation, contaminants

## Abstract

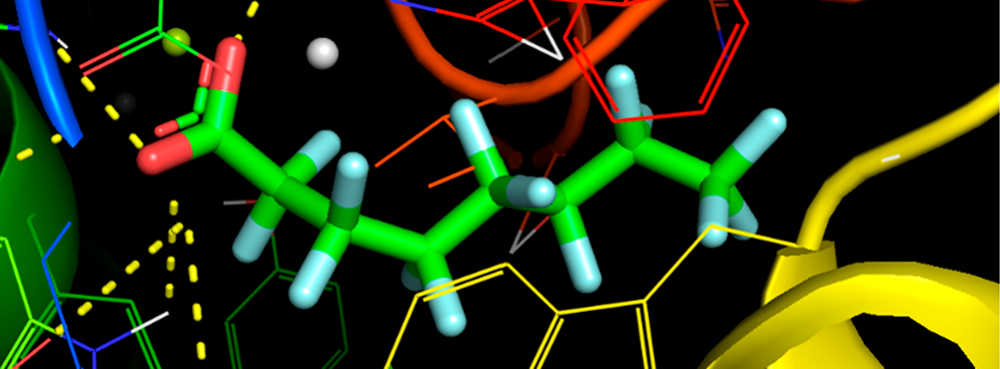

Per and polyfluoroalkyl substances (PFAS) present a unique challenge to remediation techniques because their strong carbon–fluorine bonds make them difficult to degrade. This review explores the use of *in silico* enzymatic design as a potential PFAS degradation technique. The scope of the enzymes included is based on currently known PFAS degradation techniques, including chemical redox systems that have been studied for perfluorooctanesulfonic acid (PFOS) and perfluorooctanoic acid (PFOA) defluorination, such as those that incorporate hydrated electrons, sulfate, peroxide, and metal catalysts. Bioremediation techniques are also discussed, namely the laccase and horseradish peroxidase systems. The redox potential of known reactants and enzymatic radicals/metal-complexes are then considered and compared to potential enzymes for degrading PFAS. The molecular structure and reaction cycle of prospective enzymes are explored. Current knowledge and techniques of enzyme design, particularly radical-generating enzymes, and application are also discussed. Finally, potential routes for bioengineering enzymes to enable or enhance PFAS remediation are considered as well as the future outlook for computational exploration of enzymatic in situ bioremediation routes for these highly persistent and globally distributed contaminants.

## Introduction

Bioremediation has advantages for degrading pollutants, as it can be applied on site without considerable disturbance to the environment and has the potential for cost-effective, complete destruction of the pollutant.^1^ However, per- and polyfluoroalkyl substances (PFAS) are extremely persistent with few bacteria and no enzymes yet identified as capable of fully degrading them. Enzyme engineering has been used to treat some contaminants, such as heavy metals.^2^*In silico* design has made many advances over the years with its use in industry to provide or enhance catalysis. There have been several instances of replacing natural substrates with substituted ones to achieve a desired product and/or manipulating the enzymes themselves to increase degradation rates. There has even been success in *de novo* design, repurposing or creating an entirely new enzyme for a specific purpose.^3^ However, design of enzymes to degrade the “forever chemicals” in the PFAS class has not been attempted yet. Given that no natural enzyme has been identified to degrade PFAS, an *in silico* rationally designed enzyme could revolutionize PFAS treatment.

PFAS have become an increasingly concerning environmental hazard. Many long-chain PFAS accumulate in soil, water, and humans, and are associated with myriad health effects including reproductive and developmental toxicity, immunotoxicity, hepatic and metabolic toxicity, endocrine disruption, and tumor induction.^4^ Increasing the concern is the continued widespread use of these chemicals, which have historically been found in firefighting foam, paper products, wire manufacturing, textiles, and industrial surfactants, among others.^5^ They are defined as either fully fluorinated hydrophobic carbon chains (perfluorinated) or at least one carbon not fully fluorinated (polyfluorinated).^6^ The polyfluorinated compounds are sometimes referred to as “precursor compounds” as they have been known to degrade from biotic and abiotic mechanisms into perfluorinated compounds which then do not degrade further.^7,8^ The most commonly studied PFAS in this review are the perfluoroalkyl acids, PFAAs, with either carboxylate or sulfonate head groups with varying hydrophobic chain lengths. The majority of studies reviewed here have focused on two long-chain PFAS, perfluorooctanoic acid (PFOA) and perfluorooctanesulfonic acid (PFOS).

Degradation of these compounds is difficult due to the strong carbon–fluorine bond (460 kJ/mol).^9^ Methods for degrading PFAS include thermal destruction, chemical redox, or electrochemical oxidation.^10^ However, these methods have a number of downfalls, including being chain length-specific and functional group-specific.^10^ This leads to inefficacy in combating the wide range of PFAS present in the environment. There is also concern over incomplete degradation creating short-chain PFAS, the high energy/costs, and challenges for field-scale applications.

Limited knowledge of microorganisms and biological mechanisms capable of degrading PFAS has slowed development of this promising technology. The list of bacteria known to be capable of biodegrading PFAS is short, with little research following defluorination rather than just transformation of the parent compound.^10−12^ Moreover, the enzymes responsible have yet to be identified, leading to difficulty in understanding whether these organisms can be applied to the wide range of PFAS.

The goal of this review is to evaluate the landscape for bioremediation of PFAS by *in silico* enzyme design. Currently known abiotic chemical redox methods are reviewed, focusing on their success in degrading PFAS. If we consider successful chemical redox techniques using high-energy radicals, the answer for bioremediation might lie in enzymes that use radicals to facilitate their reactions.^11,10^ Therefore, this review provides an overview of redox potentials and PFAS degradation outcomes compared to the redox potential of various metal complexes and enzymatic radicals, along with their limitations. We then identify potential enzymes that could be used for PFAS degradation, discuss the structures of these enzymes, and their reaction mechanism(s) with native substrate(s). This includes the only enzyme, to the best of our knowledge, that is well-studied and capable of breaking a carbon–fluorine bond, fluoroacetate dehalogenase.^13^ Given the many carbon–fluorine bonds present in PFAS, it seems unlikely such a natural enzyme could be a cure-all, particularly since the energy requirements differ greatly to break a bond in a disubstituted versus a tetra-substituted carbon atom. However, it may be a possible starting point for computational design. Outside this enzyme there have been some advances made in using radical-generating enzymes, such as laccase or horseradish peroxidase, to degrade PFAS.^14−17^ These more practical approaches may be amenable to enhancement. If no route with an existing enzyme seems feasible, an attempt may be made toward complete *de novo* design—building an enzyme from scratch. With this in mind, the basics of computational enzyme design, existing techniques, and current limitations are reviewed, as they may apply to radical enzymes and potential PFAS degradation. Finally, we outline a potential roadmap for multiple engineering pathways that might be used for PFAS remediation.

## PFAS Degradation

There are over 4700 chemicals in the PFAS family, and the need for remediation of both water and soil from contamination by this diverse class of chemicals is driving development of varied technologies.^5^ Thermal treatment, filtration, adsorption, soil washing, advanced reduction/oxidation processes, and bioremediation have been discussed in previous reviews.^11,10,18−20^ Existing information suggests that bioremediation, though attractive, remains ineffective when compared to more energy-intensive approaches. The redox strength of radicals and degradation routes used in redox remediation techniques may hold key information that can be applied to enzymatic degradation routes. There have been many redox systems investigated for PFAS remediation; more information may be found in reviews by Dang et al. 2020 on advanced reduction processes, Onwudiwe et al. 2020 on photoenhanced degradation of PFAS, or Alalm and Boffito (2022) on mechanisms and pathways.^20−22^ Here, we highlight the general pathways and limitations of these techniques (see Table 1).

**Table 1:**
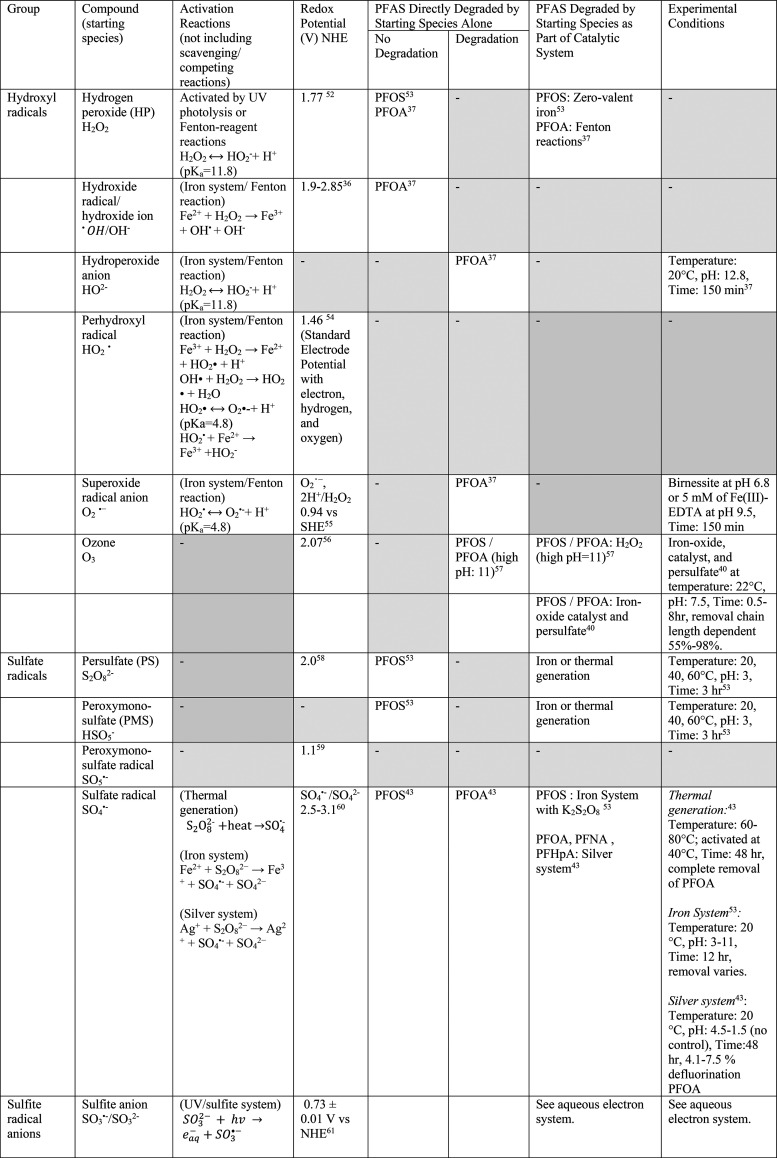

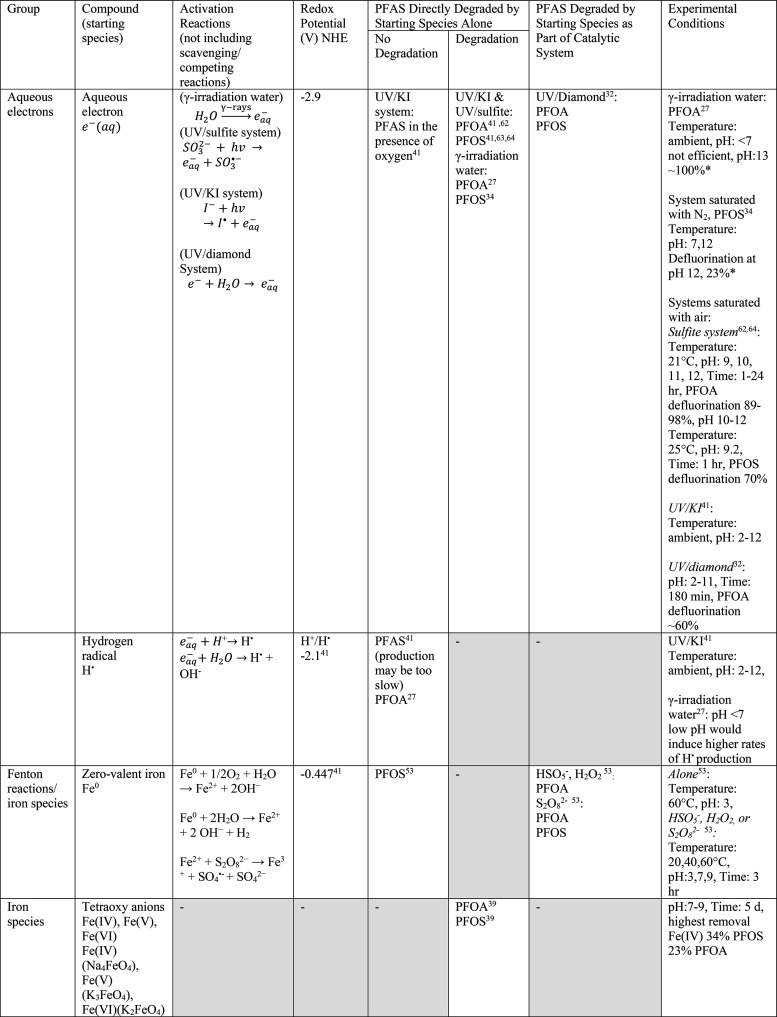

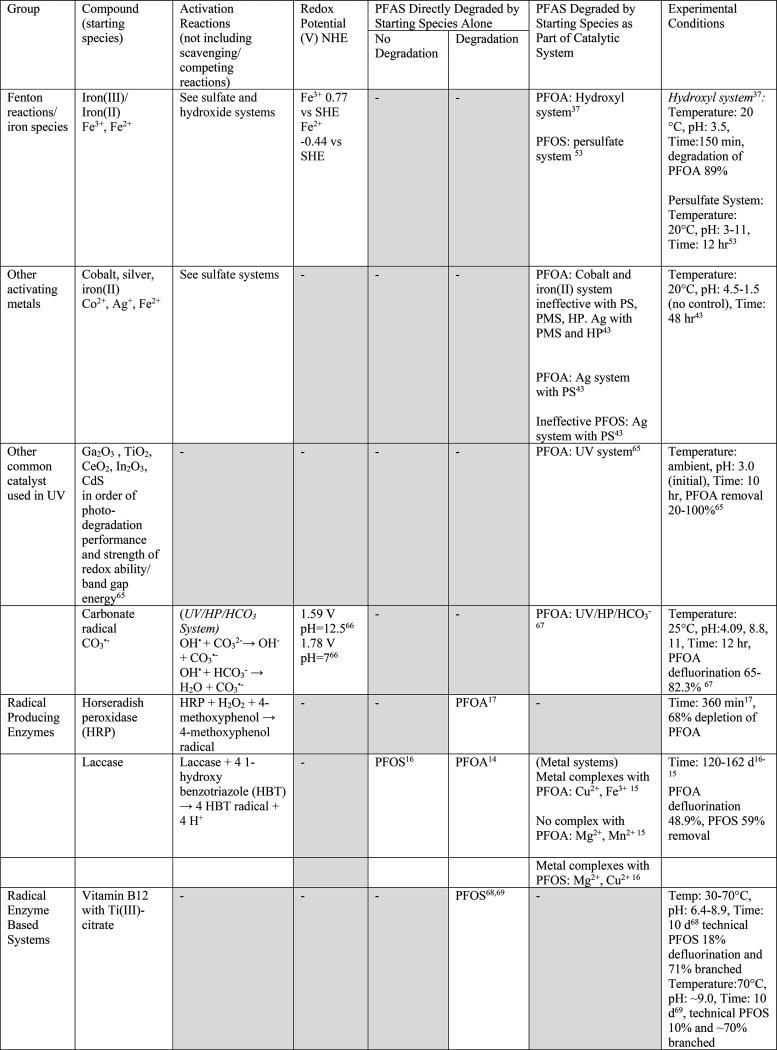
Redox Potentials for Currently Known PFAS Degradation/Adsorption Systems and Their Respective Effectiveness^a^

a Refs (52−67).

## Reductive Techniques

### Hydrated Electrons

Hydrated or aqueous electrons (e^–^_aq_) can be formed from pulse radiolysis or sonolysis of aqueous solutions, double-pulse laser excitation of water, or photodetachment from electron donors.^23^ This powerful reductant, which has been shown to degrade PFAS, has a redox potential of −2.9 V vs normal hydrogen electrode (NHE),^24^ and is usually combined with a catalyst to reduce PFAS. There is still some debate surrounding both the reaction mechanism and structure of the hydrated electron shell.^25,26^ The dominant model is the excluded-volume structure with the hydrated electron occupying a quasi-sphere cavity surrounded by H_2_O^–^ clusters. The strongest theory for the reaction mechanism for e^–^_aq_ with other molecules is that the e^–^_aq_ reacts via electron transfer. However, it has been suggested that proton transfer pathways into the cavity are also possible.^14^ The reaction kinetics involving e^–^_aq_ can be diffusion-limited when activation energies are low (1–8 kcal/mol).^23^ This is important to keep in mind, as the high-energy e^–^_aq_ will not have a long lifespan. If the PFAS molecule is far from the e^–^_aq_ or has a high activation energy, then the e^–^_aq_ may react with other compounds before reaching the target PFAS. It is also important to note that water radiolysis generates three key species: the hydrated electron e^–^_aq_, a hydrogen radical (H^•^), and a hydroxyl radical (HO^•^). Understanding how each species interacts with PFAS allows for optimizing degradation pathways.

The simplest systems to evaluate the hydrated electron’s ability as a PFAS degrader may be those created by gamma irradiation. In this type of system it is found that e^–^_aq_ will degrade PFOA through removal of the parent compound, but the accompanied HO^•^ is needed for complete destruction of the compound through defluorination, including of transformation products.^27^ The defluorination rate of e^–^_aq_ systems generated via water irradiation are also found to depend on the PFAS headgroup and chain length.^28^ Interestingly, this headgroup and chain length dependence was not found in initial steps of the reaction. Activation energies for linear perfluoroalkyl sulfonates and carboxylates were recently determined experimentally using temperature-dependent transient absorption spectroscopy. It was found that activation energies were similar, averaging 2.63 kcal/mol.^29^ This indicates, rather, that the rate is diffusion limited. This was further indicated with computational studies of e^–^_aq_ with PFOA and PFOS.^30^ Another method of e^–^_aq_ generation for PFAS degradation is via use of UV with an electron donor such as iodine or sulfite. These systems will also create I^•^ or SO_3_^–•^. While the sulfite radical is not as strong of a reducer as e^–^_aq_, much like the hydroxyl radical it is suggested that both SO_3_^–•^ and I^•^ might interact with PFOA degradation products.^20,31^ The effectiveness of e^–^_aq_ is greatly dependent on pH, due to competing reactions, where quenching of e^–^_aq_ by H^+^ is favored in acidic conditions. However, in alkaline conditions H^•^ and OH^–^ generate additional e^–^_aq_. A UV/sulfite system at pH 10 can reach 100% transformation of the parent PFOA within an hour, and defluorination can reach 89% within 24 h.^31^

The pH-dependence of this process has led to a search for a more efficient catalytic system. One example includes a UV/diamond system where the hydrated electrons are generated directly from the diamond catalyst by UV radiation and can subsequently be transferred from the surface of the diamond to the solution. The system was shown to have little pH dependence, with rates of PFOA removal ranging from 0.01823 ± 0.0014 min^–1^ at pH 2 to 0.02208 ± 0.0013 min^–1^ at pH 11.^32^ Carbonate, nitrate, and humic acid have been shown in UV/sulfite systems to diminish PFAS degradation efficiency, which is problematic because these species are likely to be found in ground or surface water.^31^ In an attempt to resolve this issue, a system that uses indole derivatives to generate hydrated electrons within a system containing humic substances was recently explored. The most efficient systems tested were the indole and skatole systems. These systems have two important qualities: one is the high amount of e^–^_aq_ that can be generated; the second is the hydrogen bonds that form between PFOA and indole/skatole and promote electron transfer between the two molecules.^33^

**Figure 1:**
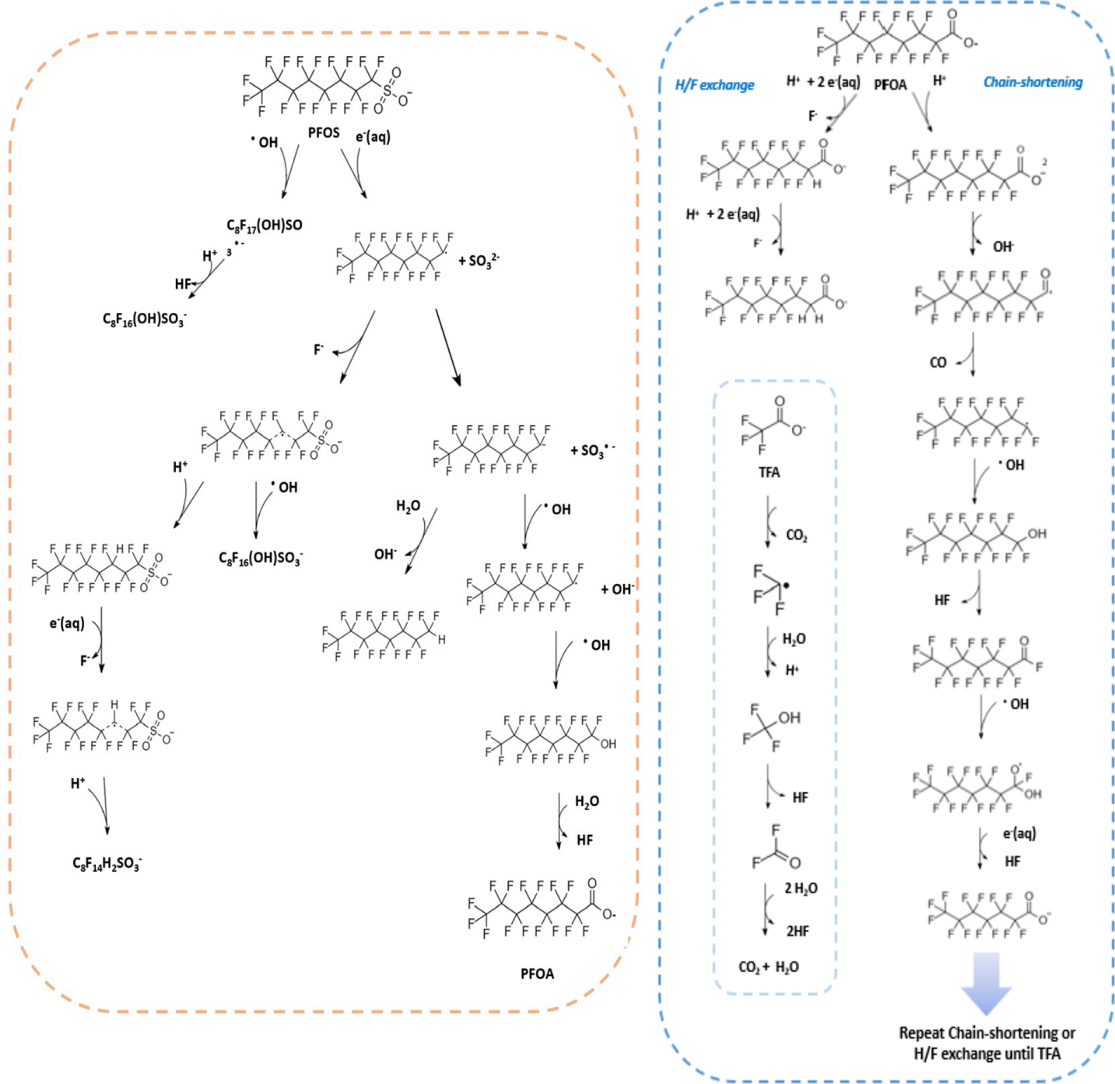
Possible route of degradation of PFOS (left) and PFOA (right) in aqueous solution with gamma irradation to generate hydrated electrons and hydroxyl radicals.^27,28,34^ The PFOS scheme was proposed to explain observed transformation products. Other reactions could continue the transformation process (for example, the PFOA product could enter cycle on the right) to result in further degradation.

Reaction pathways can become complicated due to presence of additional radicals that can interact with PFAS intermediates and smaller-chain degradation products. Figure 1 shows possible simplified reaction routes in a water irradiation system, as described by Liu et al. 2019 and Zheng et al. 2014 for PFOA and Trojanowicz et al. 2020 for PFOS.^27,28,34^ While e^–^_aq_ systems are capable of degrading both PFOS and PFOA, they are dependent on the OH^•^ concentration, can be pH and O_2_ sensitive,^27,34^ and are less effective at defluorination of PFOS. The pH limitation has been overcome with the use of diamond as a catalyst to create e^–^_aq_, but detecting free electrons from solid surfaces is still a developing approach and thus finding solid catalysts to create e^–^_aq_ can be difficult.^35^ There is also the inability to control the hydrated electron and coradicals reaction pathways. Some resolve this by flooding the system with hydrated electrons and possibly oxidizers to guarantee a reaction with PFAS; others use a catalyst that not only generates e^–^_aq_ but also makes the PFAS more favorable for reaction by forming a complex with it to either bring it closer to the radicals or lower the activation energy for C–C bond breakage and/or defluorination.

## Oxidative Techniques

### Hydroxyl Radical

The hydroxyl radical is a powerful oxidant (•*OH*/OH^–^ 1.9–2.85^36^ V/NHE) usually generated from Fenton reactions. Hydrogen peroxide (H_2_O_2_) catalyzed into radical species often undergoes propagation steps with organic pollutants as a method of remediation for contaminated environments. A study investigating the effectiveness of a modified Fenton reaction using Fe(III) and H_2_O_2_ on PFOA^37^ showed that a system containing hydroxyl radicals (HO^•^), superoxide radical anions (O_2_^•–^), hydroperoxide anions (HO_2_^–^), and perhydroxyl radicals (HO_2_^•^) could degrade 89% of PFOA in 150 min, based on removal of the parent compound. The hydroxyl radicals, though used in a variety of treatment technologies, are unreactive on their own with PFOA. Superoxide radicals and hydroperoxide anions are both capable of degrading PFOA on their own, resulting in 68% and 80% degradation of the parent compound, respectively, in 150 min. Defluorination rates for the hydroperoxide anion system indicate nearly complete defluorination of PFOA.^37^

### Metals

Metals with high oxidative potentials transform some PFAS into radical intermediates, allowing for further degradation. Iron-complexes, for example, are often found in enzymes as radical generators, so understanding their reactions with PFAS outside an enzymatic environment is of interest. Iron(III) exposed to visible light in aqueous solution will not degrade PFOA. However, under sunlight 97.8% of PFOA was degraded, with 12.7% complete defluorination, in 28 days.^38^ The tetraoxy anions Fe(VI), Fe(IV), and Fe(V) were found to serve as oxidants to both PFOS and PFOA.^39^ In this case the pH influenced the amount of residual parent product, with neutral pH favoring Fe(VI) to oxidize both compounds more effectively, while Fe(IV) showed higher PFOS degradation under alkaline conditions. Overall, the highest removal was 34% for PFOS from Fe(IV) under alkaline conditions and 23% for PFOA under neutral conditions. Defluorination rates could not be determined due to conflicting results on the presence of fluoride ions in solution; incomplete mineralization of the PFAS compounds or ion removal by the Fe(III) oxides/hydroxides was suspected.^39^

### Persulfate

Hydroxyl radicals and ozone molecules alone are ineffective in PFAS remediation. However, in combination with an iron-oxide catalyst and persulfate, ozone is effective in removing a variety of PFAS groups, where the (SO_4_^•–^) radical is believed to be generated along with other reactive oxygen species. Interestingly, PFAS chain length influenced removal rather than the functional group (carboxylate versus sulfonate) of the PFAS molecule in this heterogeneous catalysis system. PFAS chain lengths between 7 and 11 carbons had a 98% removal efficiency, 12–17 carbons had 64%, and 4–6 carbons had only 55%.^40^ This type of carbon chain length effect can be seen in both UV/Iodide and UV/Sulfate systems, where PFOS degrades more rapidly than 6 or 4-carbon PFAS.^41^ This further stresses the importance of studies that measure defluorination, since complete destruction of PFAS, rather than simply shortening the chain length to something that is harder to destroy, is the goal. Production of the sulfate radical is highly temperature dependent. The system is completely ineffective at degrading PFAS until 40 °C. Moreover, PFOS is not degraded at temperatures up to 90 °C. However, efficacy at room temperature can be improved with a catalyst. When combined with Iron(II), K_2_S_2_O_8_ at 20 °C can defluorinate PFOS, although not very efficiently when compared with other systems studied (hydrothermal and UV),^42^ and is also sensitive to pH, with more acidic conditions improving degradation.

The homogeneous persulfate/silver (Ag^+^) system was very effective in degrading PFOA, unlike persulfate/cobalt (Co^2+^) and persulfate/iron (Fe^2+^), which were not capable of degrading PFOA at 20 °C due to lower ability to generate the sulfate radical. Ag^+^ can generate sulfate radicals along with Ag^2+^, and Ag^2+^ can also react with RCO_2_H, generating a carboxylate radical (RCOO^•^) and H^+^ oxidizing silver back to Ag^+^. The hydroxyl radicals and Ag^2+^ influence PFAS degradation to a lesser extent. Much like the previously discussed systems, degradation of the parent compound favors longer-chain PFAS over shorter. Unlike the ozone/sulfate system, the persulfate/silver system is functional group dependent, and is unable to degrade PFOS. The carbon-sulfur bond is reasoned to need a higher energy source to degrade than the sulfate radical can provide.^43^ This outcome is interesting when compared with the K_2_S_2_O_8_ PFOS system. While radical generation is much lower in the K_2_S_2_O_8_/Fe(II) system, there was still some defluorination (around 23% defluorination over 12 h) indicating that the persulfate radical is capable of some PFOS degradation. However, the PFOA sulfate/Fe(II) system does not generate enough radicals to degrade PFOA.^43,42^

**Figure 2:**
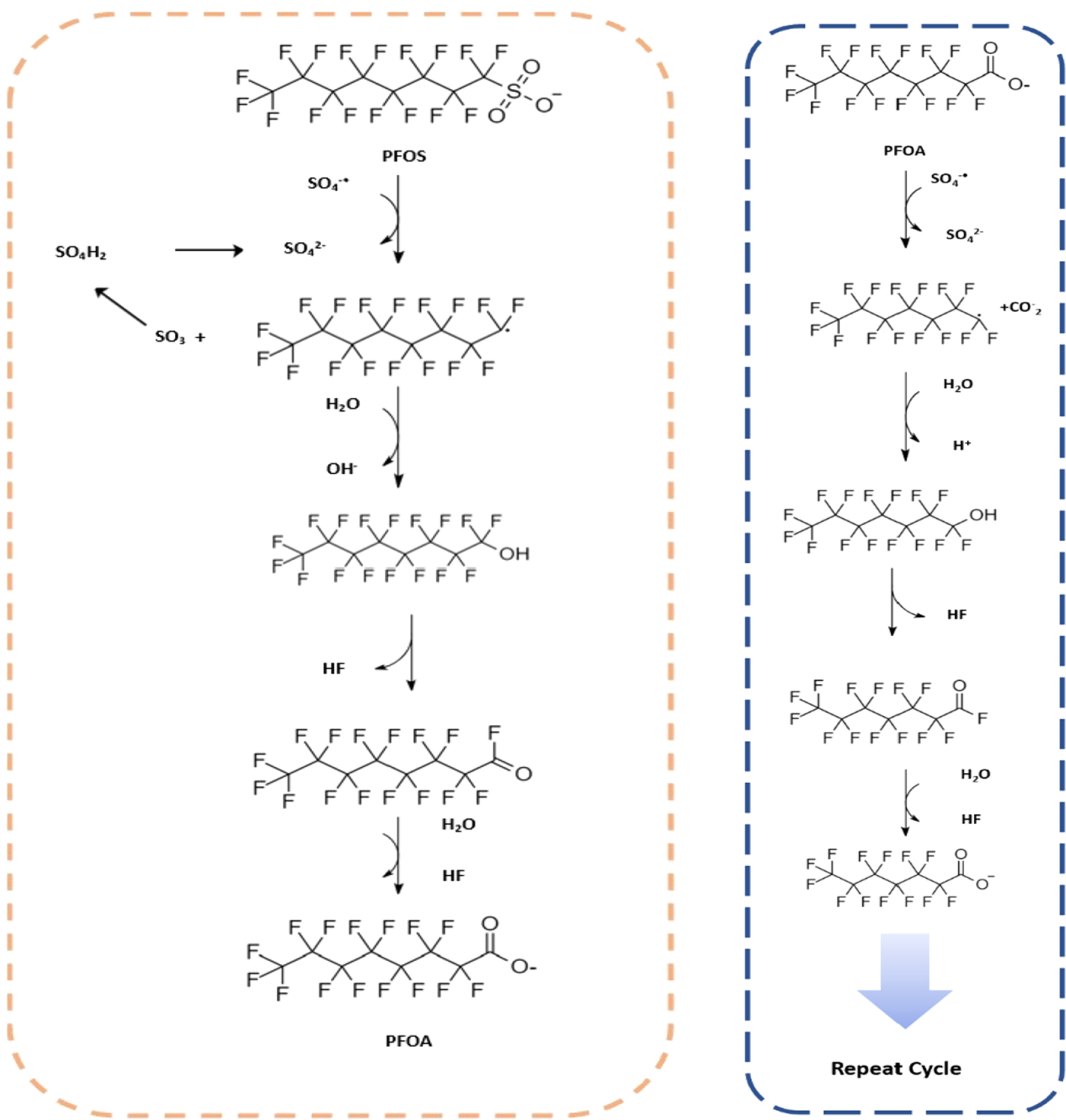
Possible routes of degradation for PFOS (left) and PFOA (right) for a UV/persulfate system.^42,44,46^ Note that in this scheme degradation of PFOS yields PFOA, which can then enter the right-hand cycle.

In the UV/persulfate system, the SO_4_^•–^ radical can degrade both PFOS and PFOA along with shorter chain length PFAS.^41^ While PFOS will degrade faster than PFOA, full defluorination of the former is harder. Defluorination has been shown to reach ∼74% after 12 h for PFOA in SO_4_^•–^ irradation systems,^44^ while PFOS reached only ∼16% over the same time.^42^ Increasing the amount of S_2_O_8_^–^ in the system will eventually lead to saturation with PFOA^44^ and PFOS^42^ degradation. This is believed to be from SO_4_^•–^ reacting with S_2_O_8_^2–^.^44^ Overcoming competing reactions by saturating the system will also cause unreasonably high energy demands when using the sulfate radical in water treatment, where complex chemical mixtures may be present.^45^ Possible reaction routes for PFOS and PFOA with persulfate are reported in Figure 2 (adapted from Cheng (2013) and Hori (2005)). The multiple reactions may be better controlled with enzymatic degradation, as enzymes can not only effectively control radicals within their activation sites but also selectively react with specific substrates.

## State of Knowledge on Enzymatic Remediation

### Microbial Degradation

Bioremediation may be a helpful tool in the treatment of PFAS. Evidence is emerging that some bacteria are capable of biodegrading these persistent chemicals, with studies indicating 32% to 75% decrease in the parent compound from biological treatment.^11,12^ For example, *Pseudomonas plecoglossicida* 2.4-D can degrade PFOS to perfluoroheptanoic acid, reducing the parent compound by 75%.^47^ Another bacterium, a strain of *Acidimicrobium* sp., has been shown to partially defluorinate both PFOA and PFOS, removing up to 60% of the parent compounds, with analysis by ultrahigh-performance liquid chromatography–tandem mass spectrometry (UHPLC-MS/MS) and ion chromatography, indicating formation of both shorter chain PFAS and the release of fluorine.^48^ However, these studies utilize highly specific organisms that are extremely slow-growing and show only partial degradation. One promising alternative may be the application of mixed aerobic or anaerobic communities containing a range of organisms capable of degrading different compounds (Zhang).^47^ Mixed communities isolated from wastewater sludge and commercially available cultures of known dehalogenators have also been shown to effectively degrade various PFAS compounds.^49−51^ Regardless of the culture used, few studies have followed the defluorination of the parent compound and thus cannot differentiate between degradation or removal via other processes (e.g., sorption). In addition, most studies fail to analyze short-chain PFAS compounds created from the parent, and thus cannot conclusively show complete removal of all PFAS.

### Horseradish Peroxidase (HRP)

The isolated enzyme HRP was one of the first to be evaluated for PFAS degradation, and has been used along with hydrogen peroxide and 4-methoxyphenol as a cosubstrate to degrade PFOA in solution. The methoxy radical produced in this system initiates a reaction cycle that creates many PFAS side products but can achieve up to 68% degradation of the parent compound.^17^ The lack of control over the radical and the complicated reaction pathway is very similar to many abiotic chemical remediation systems.

### Laccase.

Laccase, together with 1-hydroxybenzotriazole (HBT), defluorinated PFOA by as much as 28% in a mineral-buffer solution, with ∼50% of parent compound removal. The mechanism is a cycle referred to as an enzyme-catalyzed oxidative humification reaction (ECOHR). The HBT is used as a substrate for Laccase and released as a free radical capable of defluorinating PFOA. However, there are also many side products generated during this treatment cycle, owing to the radical’s short lifetime and inability to be controlled outside the enzyme, along with the distance between the enzyme and PFOA.^14^ The mineral-buffer solution was analyzed by exploring the effects of the metal ions. It was found that Fe^3+^ and Cu^2+^ can complex with PFOA and increase the HBT radical efficacy; the resulting decomposition of PFOA was similar to laccase and HBT in the complete mineral-buffer solution, while Mg^2+^ and Mn^2+^ displayed very little degradation.^15^ PFOS was also tested with metal ions, Mg^2+^ and Cu^2+^, in laccase and HBT solution, resulting in degradation of 59% over 162 days. The presence of metals that could complex with the chosen PFAS was believed to shorten the distance between the enzyme and the electronegative PFOS and PFOA, thus allowing for a higher probability for the HBT radical to react with them.^16^ Carboxylate functional groups complex better with Fe^3+^ and Cu^2+^ while the degradation of PFAS containing sulfonate groups is increased with Mg^2+^ and Cu^2+^.

Overall, enzymatic defluorination and degradation seem to work well when a catalyst is able to form a metal-complex interaction, bringing PFAS closer to the enzyme, as well as creating the reactive species of reductant/oxidant. This can help overcome pH, structure, headgroup, and chain length dependence depending on the system/complex. The proximity issue, in particular, is a problem in known engineered bioremediation techniques, due to systems incorporating enzymes that release the radical into a solution rather than having it react within the enzyme, unlike what happens in most natural radical enzymes.^17,14−16^ Thus, a helpful a starting approach to system design may be to evaluate various redox potentials and their effects on PFAS, along with the system catalyst. PFAS begin oxidizing at around 2.0 V NHE and reducing at <−1.1 V NHE.^41^ This seems to lead to the conclusion that PFAS reduction is more thermodynamically favorable. However, defluorination may require oxidation, as evidenced by the hydrated electron system. Redox potentials change in relation to environmental conditions, and thus a system with a low or high redox potential in the gas phase may work differently within the enzymatic environment, much like how hydrogen peroxide works in conjunction with zerovalent iron. Therefore, understanding the metal and substrate’s redox potentials within an enzymatic environment is needed. Investigating which metals complex with PFAS to encourage radical degrading reactions would also be useful for design. While the base redox potential of enzymes and their metal-complexes may seem too low to enable PFAS degradation, the enzymatic environment helps facilitate these reactions.

## Possible Enzymes for PFAS Biodegradation

With a need to identify bacteria or enzymes capable of effectively degrading PFAS and little information on enzymes that naturally degrade PFAS we turn our attention to redox reactions facilitated by high-energy radicals and fluoroacetate dehalogenase (FAcD). The previous section focused on chemical redox techniques that created radicals within their systems to break the C–F bond. It follows that an enzyme capable of breaking the C–F bond may be found among the radical generating enzymes. These include the well-known enzymes, *S*-adenosylmethionine (SAM or AdoMet) and Adenosylcobalamin (B12)-containing enzymes, horseradish peroxidase (HRP), and laccase. Several reviews have been written on the mechanisms of radical enzymes, particularly SAM,^77−79,72^ B12,^77,80,72^ and dehalogenases.^81^ Here we briefly describe FAcD, along with a few of these radical-incorporating enzymes with a focus on those that might be of interest for PFAS remediation and provide some guidance for further optimization and design.

### Peroxidases

As discussed earlier, HRP is an oxidoreductase enzyme frequently used to generate radicals that will further react with desired compounds in bioengineered reaction pathways^82^ and it has been used to degrade PFAS with generated hydroxyl radicals. In the environment, peroxidases are used to prevent hydrogen peroxide (H_2_O_2_) and other reactive oxygen species from harming cells. In bioengineering, it is frequently used with NADH to form hydroxyl radicals (HO^•^). The hydroxyl radical is highly reductive and can be further used to catalyze reactions. However, it is not the only radical capable of being generated by HRP.

**Figure 3:**
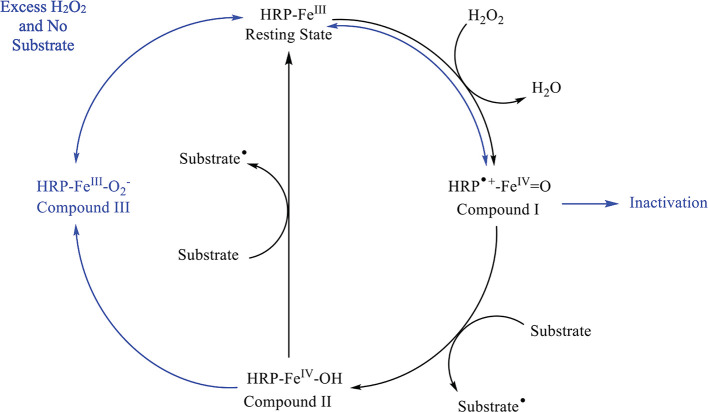
Reaction cycle of HRP in the presence of a suitable substrate and H_2_O_2_ and without a substrate and excess H_2_O_2_. The black arrows represent the typical pathway, while the blue arrows represent other possible outcomes with excess H_2_O_2_ and no substrate present. The resting state of HRP is transformed to compound I through H_2_O_2_. Here the reaction can cycle to compound II or, if no substrate is present, can deactivate the enzyme (P670), return to the resting state, or form Compound II. Compound II will either return to the resting state (substrate/excess H_2_O_2_ pathway) or form Compound III if excess H_2_O_2_ is present. Compound III will then return to the resting state.^83,82,84^

The natural mechanism of HRP revolves around the heme porphyrin (Por)-FeIII complex being oxidized by H_2_O_2_ and reduced by a substrate capable of donating an electron, such as phenol, as illustrated in Figure 3 below.

The ability of peroxidases to break C–F bonds seems to be limited by their substrate, H_2_O_2_. This was observed in a study involving heme haloperoxidases and nonheme halogenases. Heme haloperoxidases are known to form an iron(IV)-oxo cation radical species and nonheme halogenase species can also react via a halogen radical. Tetrabutyl ammonium halides (TBA-X) were used to study the reactivity and mechanistic pathways of nonheme iron(III)-hydroperoxo intermediates and compared to the kinetics of iron(IV)-oxo complexes. It was found that the TBA-F was unable to release fluorine ions.^85^ As discussed earlier, 4-methoxyphenol can be the electron-donating cosubstrate and its resulting radical has been used to degrade PFOA. This is one design path to optimize the radical substrate that would degrade PFAS. However, the distance of the radical substrate to PFAS will still be an issue, as discussed by Luo Qi, et al. 2015, 2017 in their work with laccase.^14,15^ This issue could be resolved by keeping the radical within the enzyme, as with B12.

### B12 Incorporating Enzymes

**Figure 4:**
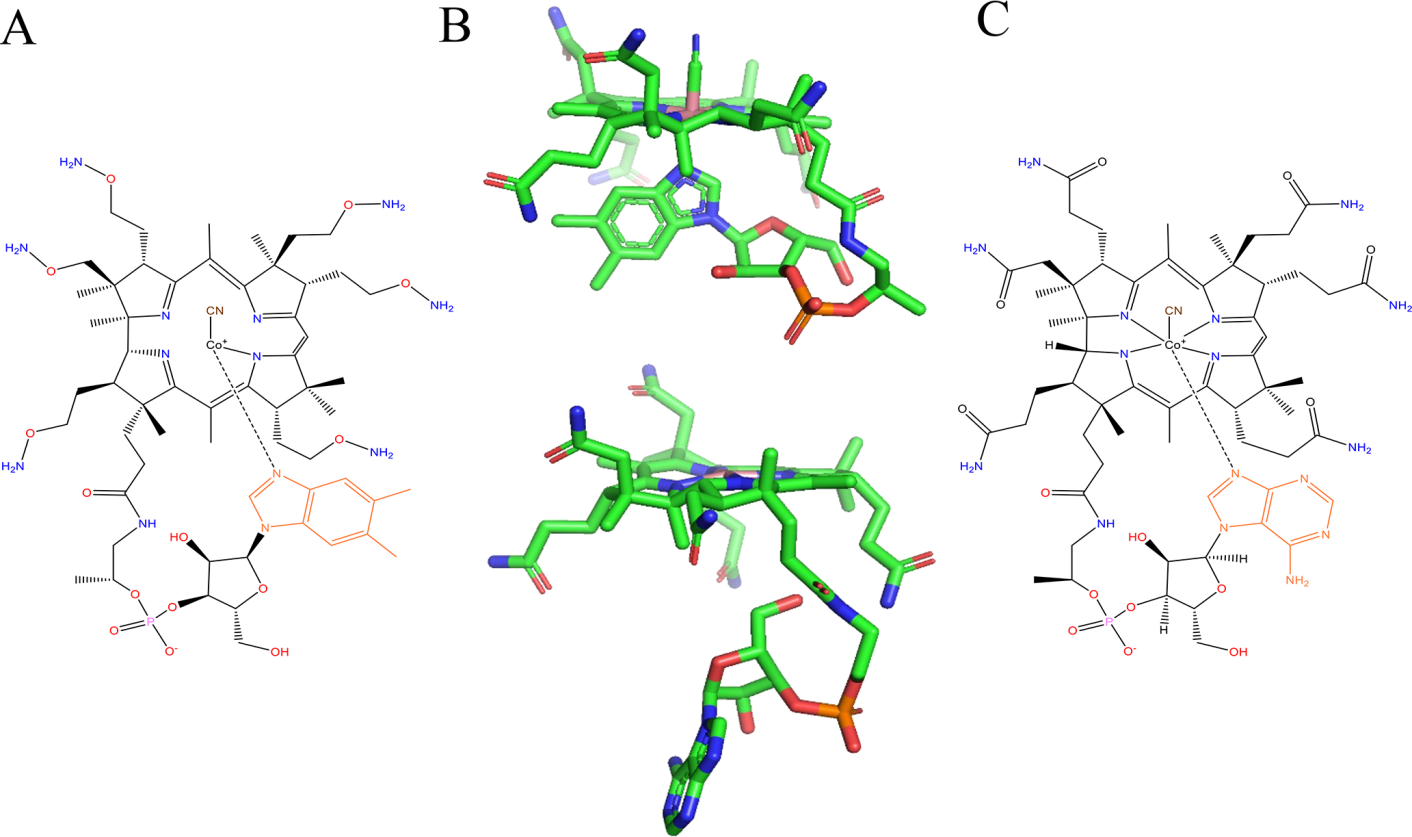
Molecular structure of cofactor B12. (A) The structure of B12 in the “base-on” form with cyanide (CN) as the R group, CN may be replaced with CH_3_ OH or 5′deoxyadenosyl. (B) B12 in the “base-on” form, top, and “base-off” form, bottom. (C) The structure of norpseudo-B12 in the “base-on” position.

B12 molecules consist of a corrin macrocycle and a dimethylbenzimidazole (DMB) ligand with a cobalt center (cobalamin). The four nitrogen atoms on the macrocycle tightly surround the inner cobalt while also creating a flexible chain for the DMB to be on the lower alpha face of the cobalt center^80^ (Figure 4A). The uniqueness of the B12-dependent radical enzyme versus the other enzymes discussed thus far is the cofactor containing both the metal catalyst and what would, in previously discussed enzymes, be the substrate radical. The B12 keeps its radical in a stable form until it is necessary for a reaction.^77^ Enzymes containing B12 as a cofactor (e.g., tetrachloroethene (PCE) reductive dehalogenase, PceA and Nitratireductor pacificus pht-3B catabolic reductive dehalogenase, NpRDha) are considered potential PFAS degradation enzymes due to the metal-complex’s ability to act as a dehalogenator. The B12 geometry within these enzymes is described in two positions: the “base-on” or “base-off” forms (Figure 4B).^77,80^ AdoCbl will catalyze carbon skeleton mutases, aminomutases, or eliminases.

**Figure 5:**
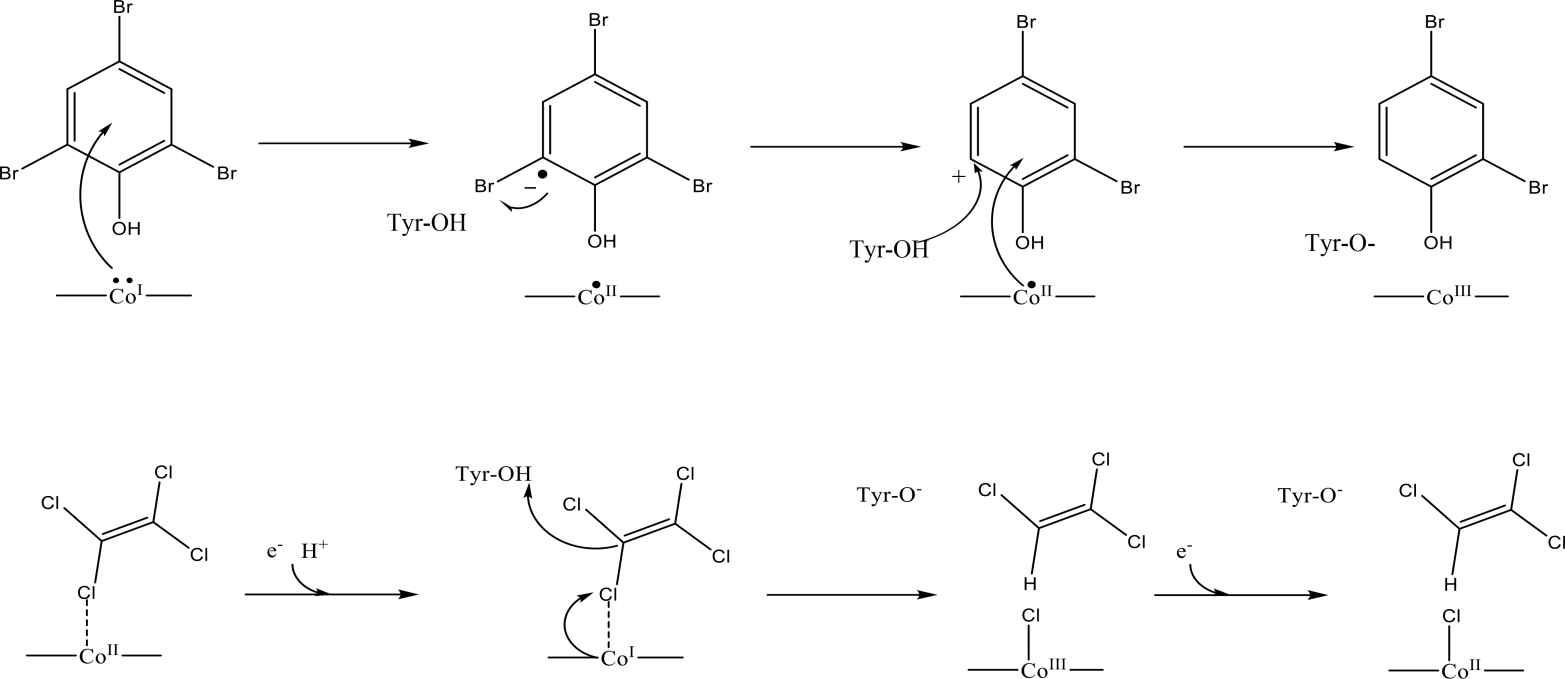
Possible dehalogenation mechanism for PCeA for brominated phenols (top) and chloroethylenes (bottom). In the first step of the bromophenol (2,4,6-tribromophenol) pathway an electron is believed to be transferred from the super-reduced Co[I] to the ring. The substrate radical is neutralized and bromine is eliminated with another electron transfer from Co[I] or an iron–sulfur cluster. A proton can be provided from the upper cavity, from a Tyr residue (shown to lower left of the phenol), or from other solvent molecules. This process will continue until 2,4,6-tribomophenol is completely dehalogenated. In the dechlorination of chloroethylene (bottom row), the mechanism is believed to follow a heterolytic pathway.

Perhaps more interesting than the radical generating cosubstrate AdoCbl prebinding to the cobalamin is the ability to substitute it with a halogen. B12 enzymes fall into dehalogenases, AdoCbl, or methyltransferases. The dehalogenating mechanism in NpRdhA is believed to be cleavage of the halogen on the substrate via binding directly to the Cobalt in the Cob(II)alamin complex, either heterolytically or homolytically (Figure 5).^75^ NpRdhA is unable to defluorinated fluorine, however it is capable of breaking the C–Cl bond.^86^ In PceA, another reductive dehalogenase containing norpseudo-B12, it was shown that a Tyr residue plays an active role in reduction of Cob(II) to Cob(I), which in turn binds to a chlorine on a trichloroethylene substrate, transferring a proton to complete the reaction.^87^ A thorough investigation by Kunze, Bummer et al. in 2017 pointed to long-range electron transfer that creates radical intermediates in PceA for brominated phenols.^88^ Liao et al. 2016 determined that the enzyme–substrate complex effected dehalogenation through a heterolytic pathway on chloroethylenes.^87^ The cob-halogen intermediate seems to be capable of forming outside of the B12 dehalogenase class. It was discovered while investigating the thiol oxidase side reactions that the *Caenorhabdiitis elegans* CblC (ceCblC) was able to form a chlorocob(II)alamin intermediate due to the presence of potassium chloride.^89^ This discovery points to possible dehalogenation properties of the AdoCbl class as well. While defluorination has not been shown, vitamin B12 has been used to degrade PFOS in a biometal system containing Ti(III)-citrate.^68,69^

### SAM Incorporating Enzymes

**Figure 6:**
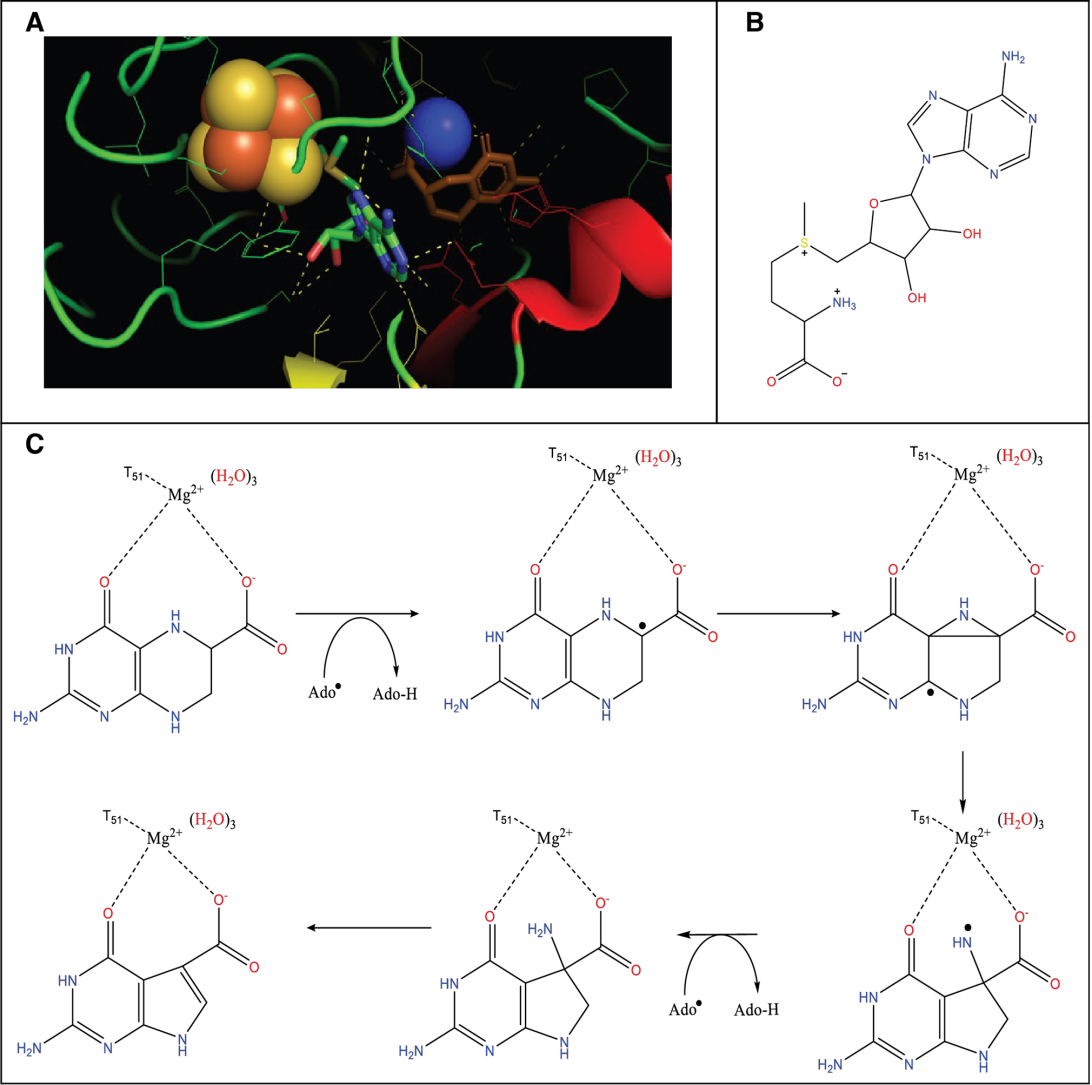
(A) The binding of SAM in the active site of QueE to the iron–sulfur cluster (orange and yellow) with the magnesium(II) ion in proximity (blue atom). (B) The structure of AdoMet. (C) The mechanistic pathway of 7-carboxy-7-deaguanine synthase by the enzyme Que. Hydrogen abstraction is followed by radical rearrangement, which is facilitated by the magnesium ion.

Enzymes that contain SAMs are very similar to B12 enzymes but are more popular in bioengineering due to their large superfamily and capability to catalyze a diverse set of chemical reactions.^84^ SAM is a sulfur-containing cofactor or cosubstrate that undergoes reductive cleavage while bonded to the cubic iron–sulfur cluster [4Fe-4S]^+^ (Figure 6). The adenosyl radical, 5′-deoxyadenosyl bonded to methionine, collectively referred to as the AdoMet radical, is generated within the active site and will dehydrogenate the substrate. The AdoMet radical will go on to induce a wide variety of chemical reactions including but not limited to amino acid conversion, epimerization, methylation, rearrangement, and carbon–carbon bond formation.^72,78^ The activation scheme of SAM is the same family wide, relying on an iron–sulfur cluster. However, the way it contains this cluster varies between family members. The metal cluster and motifs are considered a key part of controlling the AdoMet radical and coordinating the [4Fe-4S]^+^ cluster. These motifs have been described in depth in previous works.^77,72^ Recently the role of a metal ion, Mg^2+^, in the 7-carboxy-7-deazaguanine synthase (QueE) SAM enzyme was investigated.^79,90^ It was shown that the ion plays an important role in destabilizing and controlling the substrate radical. The SAM superfamily are also not limited to single metal clusters.^78,91^ A subclass of ribosomally synthesized and post-translationally modified peptides (RIPPs) contain a SAM and a SPASM domain which hold multiple iron–sulfur clusters. SAM enzyme’s ability to manipulate and control the radical is incredibly valuable for remediation.

### Fluoroacetate Dehalogenase (FAcD)

**Figure 7:**
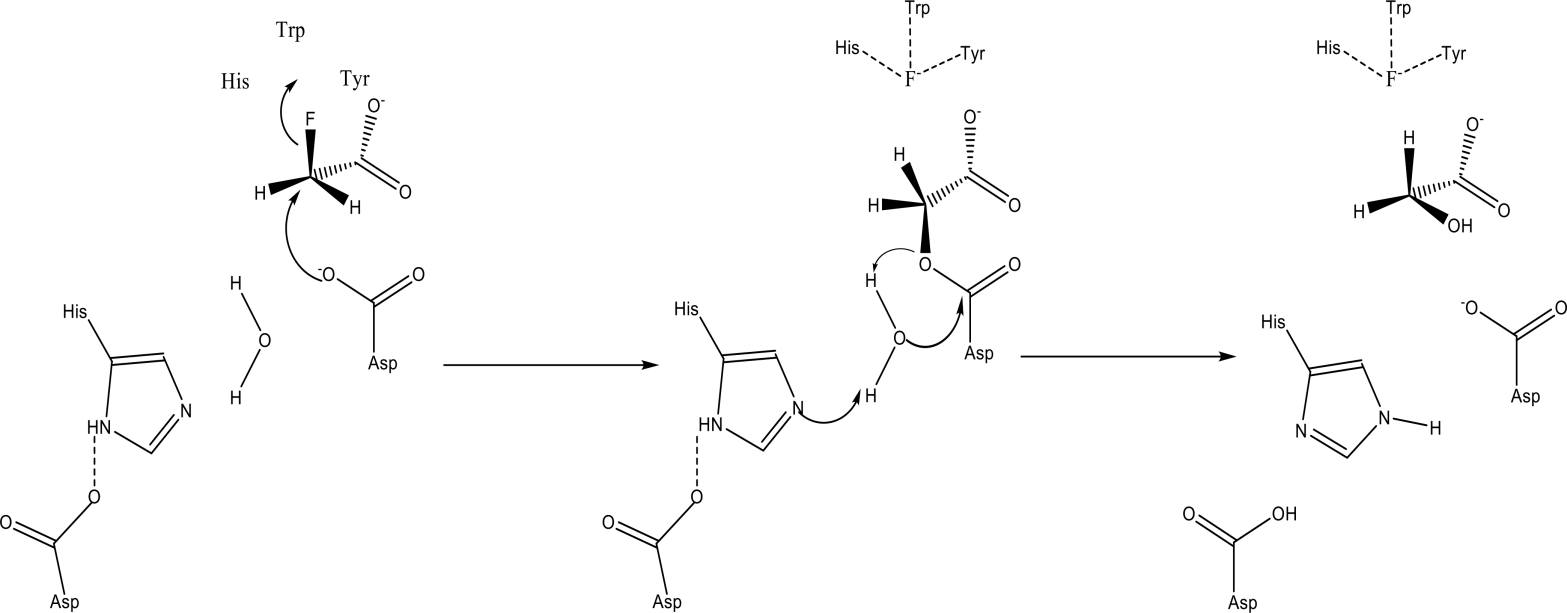
Defluorination pathway of fluoroacetate by FAcD. In step one the S_N_2 reaction takes place defluorinating fluoroacetate. In step two, water hydrolyzes the ester intermediate.

FAcD is the only well-studied enzyme capable of breaking carbon–fluorine bonds. FAcD has the ability to defluorinate both fluoroacetate and difluoroacetate by directly catalyzing C–F cleavage through an S_N_2 reaction. In the first step a key residue, Aspartate, acts as a nucleophile attacking the alpha-carbon on the substrate (Figure 7). While fluorine is a poor leaving group, the halogen-accommodating pocket in FAcD, consisting of a His, Trp, and Tyr, help to facilitate the reaction. In particular, Tyr helps to reduce electronic steric repulsion as a charge acceptor.^92^ It has been found that mutating the Trp residue will cause inactivation toward fluoroacetate.^93^ In step two, the ester intermediate is hydrolyzed by a water molecule activated by an Asp-His residue in the pocket.^92^

The C–F breakage is limited by the S_N_2 mechanism, as nucleophilic attack is hindered even in an enzymatic environment when the poor leaving group of fluorine is present in a multisubstituted carbon. Trifluoroacetate is unreactive because the activation energy for nucleophilic attack of the C–F bond steadily increases with increasing fluorination, becoming too high for reaction with trifluoroacetate.^94^ However, because of its ability to degrade short-chain partially fluorinated compounds, this enzyme shows promise for secondary substituted carbons created as side products from PFAS parent molecules.

A promising group of enzymes specifically for fluoroaromatics includes class I benzoyl-coenzyme A reductase (BCR), 1,5-dienoyl-CoA hydratase (DCH), and 6-oxo-1-enoyl-CoA hydrolase (OAH), produced by the nitrifying bacterium *Thauera aromatica*, identified for breaking down para-substituted fluoroaromatics. The reaction chain starts with BCR followed by DCH and OAH, or by two rounds of OAH, depending on the degradation pathway for complete degradation of 2-F-benzoate to CO_2_ and HF.^95^ It is important to note that in this system BCR is not involved in the C–F cleavage, but instead defluorination is catalyzed by the promiscuous DCH and OAH enzymes. The mechanism of this previously unknown mode of C–F bond cleavage catalyzed by enoyl-CoA hydratases/hydrolases was made clear by analyzing the formation of reaction intermediates using liquid chromatography/mass spectrometry (LC/MS) (Tiedt et al, 2017).^96^ Characterization of intermediates formed over the degradation pathway is critical to fully understand the roles of each enzyme in the complex mechanism of bond activation and cleavage.

## Radicals Stabilized Via Amino Acids

Amino acids can stabilize radicals either within the protein itself or by transferring electrons out of the enzyme. They are frequently found as supporting groups within dehalogenating species. Tyrosyl,^77,77,97−99^ Cysteinel,^77^ and Tryptophan^100^ have all been found to stabilize radical intermediates, we refer the reader to those papers for further information. Their respective radical redox potentials are listed in Table 2.

**Table 2:** Redox Potentials of Various Metal Centers, Active Sites, Co-/Substrates, and Radical Incorporating Enzymes

Enzyme	Redox Potential (mV)^70,71^	Metal-Complexes and Substrate/Co-Substrate^70,72−74^	Redox Potential (mV)
Horseradish peroxidase	880–900 (SHE)	3,4-dichlorophenol (not dechlorinated by NpRdhA B12)^75^	520 (SCE)
Cytochrome *c* peroxidase	740–1080	3,5-dichlorophenol (not dechlorinated by NpRdhA B12)^75^	700 (SCE)
*A. Ramosus* Peroxidase	915–1180	2,6-dicholorphenol (dechlorinated by NpRdhA B12)^75^	620 (SCE)
Myeloperoxidase	970–1350	AdoMet	–990 to −1800
Lactoperoxidase	980–1140	base-off H_2_O-cob(III)- alamin/cob(II)alamin	510
Eosinophil peroxidase	1100	base-on H_2_O-cob(III)- alamin/cob(II)alamin	200
Versatile Peroxidase	1370	Cytochrome	–400–400 (SHE)
Soybean Peroxidase	950	Iron–Sulfur Centers	∼−690–450 (SHE)
Amino Acid Radicals^76^	-	Copper Redox Centers	∼ 190–750 (SHE)
Gly	1220 (NHE, pH = 10)	-	-
Cys	1330 (NHE)	-	-
Tyr	930 (NHE)	-	-
Trp	1010, 1050 (NHE)	-	-

## Enzyme Design for PFAS Remediation

The combined complexity of both radicals and enzyme dynamics creates a unique challenge for rational enzyme design for PFAS. In the previous section, general characteristics of promising enzymes were discussed. Here, we summarize key knowledge and techniques for rational design towards PFAS remediation. We first break down the relevant radical/redox reactions and electron transfer catalysis. We then discuss techniques to design key interactions among substrates, radicals, metal-complexes, active sites, and enzymes, followed by bioengineering techniques of possible use for PFAS remediation. Finally, we discuss needs for radical enzyme engineering and specific limitations in relation to PFAS remediation.

### Radical Molecules and Electron Transfer

Several in-depth reviews provide useful understanding of radicals, stabilization energies, cycles, and energy barriers.^101,102,71,70^ Electron tunneling is a phenomenon used extensively in industry (e.g., for semiconductors) and is also found biologically in metalloproteins such as the SAM enzyme.^103,100^ Many metalloproteins create extremely reactive intermediates, and it can be perplexing how these intermediates do not react to create side products or destroy the enzyme itself. Understanding electron tunneling can help with effective mutation of sites by understanding its roles in preventing reactions or modifying rates. For more information on electron tunneling in and outside of enzymes we suggest Winkler and Gray (2014), Wenger (2011), and Gray and Winkler 2021.^103,104,100^

### Redox Potentials

Factors that influence the redox potential of a molecule or complex include pH, temperature, electronegativity, ionic strength, geometric effects, and solvent effects. In silico design requires knowledge of which factors can aid in fine-tuning redox potentials for a specific purpose. Particularly the effects of the first^71^ and second coordination spheres^105,106^ can help to understand and manipulate redox potential. The first coordination sphere consists of the metal and the covalently bonded ligands. The second coordination sphere includes the substrate and residues that have long-range electrostatic interactions with the metal. Long-range interactions, including hydrogen bonding, charge stabilization, salt bridges, and substrate positioning, can affect thermochemical properties, redox potentials, and p*K*_a_ values of residues. Iron is the most common metal in living organisms, followed by zinc. Other metals include copper, manganese, vanadium, magnesium, cobalt, molybdenum, tungsten, and nickel. The metal complex ligands play important parts in controlling reduction potentials.

### Redox Pathways of PFAS

PFAS transformation, as discussed above, can occur via the reduction route (Figure 1) or the oxidative route with a sulfate radical (Figure 2). The systematic analysis by Alalm and Boffito shows the complex route of PFAS redox pathways, with both the reactive species and the particular PFAS affecting the degradation route. Reductive routes mainly cleave the C–F bonds, while oxidative routes can break C-functional group bonds (C–CO_2_^–^,C–SO_3_^–^), C–C bonds, and C–F bonds. Heterogenous systems may weaken the functional group bond using a catalyst, while homogeneous systems use radicals, holes, and electrons to degrade PFAS.^21^ The weakest C–F bond may be the one adjacent to the terminal carbon, according to bond dissociation energy (BDE) studies,^107^ and may therefore be a good starting point for defluorination attempts.

## Enzymatic Design Techniques

The high computational demand of simulating many atoms makes it impossible to model an entire enzyme at a highly accurate quantum mechanical (QM) level. Even simulations via molecular dynamics are too costly to run for the life of many radicals, which can have 100 ns to 1 μs lifetimes.^102^ As such, studying the desired mechanism outside the enzyme can be an effective way of evaluating possible mutations and pathways. Once the enzymatic environment is taken into account, computational design becomes far more complex. This field is ever evolving and a number of reviews discuss current methods,^3,108^ but few discuss the implications when radicals are involved. This section will touch on recent computational techniques for rational enzyme design applicable for the enzymes discussed previously as well as possible *de novo* design routes for PFAS remediation.

### Enzymes without Crystal Structures

Some enzymes are extremely hard to study experimentally, such as those that are sensitive to oxygen. These enzymes usually do not have well documented crystal structures, making rational *in silico* design difficult, as the active site and surrounding residues are unknown. For example, some potential PFAS degrading bacteria, such as *Acidimicrobium* sp. strain A6, anaerobically degrade PFAS, but without a well-known enzyme structure, a route for synthetic design becomes difficult and many enzymes with promising reactions are often overlooked. However, there are some techniques that could work around the absence of an established crystal structure. The GRE BSS enzyme radical is highly reactive to oxygen and has no available crystal structure. When crystal structures are not known, the amino acid sequences can be used to predict the structure by comparing it to similar proteins, known as homology modeling. A multicomputational study that combined homology modeling, docking, molecular dynamics simulations, along with potential mean force and binding calculations was used to build the GRE BSS enzyme and evaluate the active site and influence on the radical.^98^ With this predicted structure the authors were able to propose potential binding pockets, active residues, and reaction mechanisms.

### Substrates

The specificity of enzymes makes it challenging to determine if a target structure (e.g., a specific PFAS) is similar enough to the native substrate to be catalyzed. Calculating potential energy surfaces and reaction kinetics are popular methods for substrate analysis. For example, a study on the GRE BSS enzyme was done to determine its potential for biodegradation of alkanes.^99^ A gas-phase potential energy profile for each step of the mechanism was created using the radical and toluene (a known reactive substrate) and compared to butane. The reaction kinetics were calculated using reaction rate theory and thermodynamic data. It was concluded that the desired product would react around 100 times slower than toluene and showed the importance of the substrate-enzyme complexes on reaction barriers.

### Mutating Residues in the Active Site

One of the main goals of rational design is to strategically influence the catalytic activity of a mechanism. Mutating the active site often involves a mix of computational and laboratory experiments. A guaranteed method that is completely computational has yet to be discovered, but some show promise. In one study, Empirical Valence Bond (EVB) activation energy calculations were done on a wild type (WT) dehalogenase enzyme and several mutants. The activation energy of the transition state for the substrate was calculated, while exploring the effects of key residues in the active site to first reduce the activity of the enzyme and then reactivate the enzyme using different residues at rates close to the original. This enzyme reaction initiates via an S_N_2 mechanism, much like that of FAcD, having a nucleophilic attack of a 1,2-dichloroethane releasing chlorine. With experimental data and a well-studied mechanism combined with EVB and MD trajectories, the authors were able to show experimentally that one of the two expressed enzymes showed the desired results while the other was inconclusive.^109^ EVB is often used for active site mutagenesis as a screening tool for new substrates due to the use of valence bond theory which allows for many mutants.^110^

The use of protein energy landscape exploration (PELE) is another method to further analyze the active site. This method involves predicting protein interactions with perturbations based on ligand and protein structures. It was recently used with the enzyme laccase as a first step in sampling around the T1 copper for structures by scoring mutated residues based on the amount of spin density with QM-MM calculations. The spin densities are used to estimate the electron-transfer pathway. This, combined with solvent-accessible surface area and donor–acceptor distance, was used to evaluate the results from a previous study using laboratory evolution that increased the turnover number with the substrates 2,2′-azino-bis(3-ethylbenzothiazoline-6-sulfonic acid) (ABTS) and 2,6-dimethoxyphenol (DMP). The computational study showed that the enzyme had increased turnover by improving the ligand binding more than by changing the redox potential in T1 copper.^111^

A combination of molecular dynamics simulations and QM/MM methods were used to evaluate the reactivity profile for FAcD. MD simulations were run to generate conformations for further QM/MM analysis. Potential energy profiles were then created for fluoroacetate, difluoroacetate, and trifluoroacetate along the reaction pathway, while including key residues from the active site. The reaction mechanism was broken into four elementary steps, C–F activation (stage I), nucleophilic attack (stage II), C–O bond cleavage (stage III), and proton transfer (stage IV), and generated energy profiles for each. The rate-limiting step was determined to be C–F activation (stage I) for difluoroacetate and trifluoroacetate but nucleophilic attack (stage II) for fluoroacetate. Bond dissociation energies were also calculated to compare the energy barrier and bond length of each substrate’s transition state for the rate determining step. Distortion and interaction analysis was used to evaluate the influence of the Asp residue on the tested substrates, finding that the distortion energy increases as carbon–fluorine substitution increases on the substrate. Lastly, a distortion-interaction model that sampled the electrostatic influence around the active site helped theorize which residues would influence C–F bond activation and could possibly be mutated to allow for defluorination of more heavily substituted substrates.^94^ The analysis showed that Tyr had the greatest impact on the rate-limiting step for di- and trifluoroacetate. As stated earlier, Tyr normally plays an important part by acting as a charge receptor during the S_N_2 reaction step for fluoroacetate.^92^ While the halogen pocket is believed to be an important factor in lowering the activation energy for the S_N_2 reaction for fluoroacetate^112^ mutation of other surrounding residues could help lower the activation energy required for defluorination of poly- and perfluorinated molecules.

Radicals. Many biocatalyst mechanisms seek to apply an enzyme’s capability to a different substrate. To do this, the molecule that generates the radical may be changed, as was done with laccase using HBT as the substrate radical to degrade PFAS. Recently, a study on the SAM QueE enzyme used RSE to evaluate radical rearrangement and the effects of the metal ion, Mg^2+^, on stabilization and control. The relative energies were calculated for radical rearrangements, substitutions, and complexation reaction barriers, energy, and RSE of several different metal ions in the gas phase. On the basis of experimental evidence that surplus Mg^2+^ increased the kinetics of the reaction, the energy barriers of the radical and Mg^2+^ ion were explored. It was shown that the SAM QueE enzyme relies on the Mg^2+^ ion not only to help create the radical by placing QueE into the optimal position for reduction, but also to control radical rearrangement and stabilization.^90^ Jager et al. furthered this research with a computational study in which the counterion-complexed models were able to lower energy barriers as well as radical stabilization energies. They performed theoretical calculations on rearrangement barriers, RSEs, and reaction energies when substituting the Mg^2+^ ion, revealing that out of Na^+^, K^+^, Ca^2+^, and Mn^2+^ only Mg^2+^ and Mn^2+^ exhibit a negative reaction barrier and Mg^2+^ also had the lowest rearrangement energy. This continues to highlight the significant specificity of proteins with metal ions and radical intermediates even outside the enzymatic environment.

## Metals

The metal centers within metalloenzymes are key to catalytic activity. Recent reviews on metal-coordination spheres and their effects on enzymatic activity^106,113−115^ discuss how manipulation of the metal can increase redox potentials, allowing for different substrates or cofactors to be used within an enzyme or changing the rates of reactions.

### Fine Tuning with Metals and Residues.

In the B12 enzyme, within the first coordination sphere is DMB, which affects the geometry of the cofactor by switching it into the on/bound or off/unbound position as discussed previously. Comparable to the inactive-redox metals, the DMB affects the redox potential and metalloprotein activity of the cluster, with various enzymes requiring either the “on” or “off” form for activity. The ability to change the redox potential of complexes even further would be useful for the high redox potential requirements of PFAS. One study showed that the reactivity of a single protein could be fine-tuned over the range of 0.97 V to −0.954 V vs SHE with five mutations of residues and two of the metal ions.^116^ The study used an azurin protein with a copper complex, which is similar to the metal complex found in laccase. Much like in previously discussed techniques PES calculations can be done to assess the likelihood of features such as conformation or redox state using energy profiles of metal-complexes in gas-phase calculations.^117,118^ In-depth review of these techniques is beyond the scope of this paper; the method/basis set along with solvation model should be investigated thoroughly for the system of choice.

### Valence Metals in Enzymatic Sites

Replacing metals in metal-complexes can also be used to affect reactivity. An example of this is research done on the catalytic cycle of Mn_4_CaO in Photosystem II. Synthetic clusters of Mn_3_[M]O_2_ where M equals Mg^2+,^ Ca^2+,^ Zn^2+,^ Sr^2+^, and Y^3+^ with known redox potentials were studied with QM simulations to see the effect of redox potential on replacing nonredox metals. The study concluded that the valence of the metal mainly affects redox potential rather than the ionic radius of the metals. However, Mn_4_CaO does not show a great loss in redox potential when Ca is substituted out, unlike the artificial clusters. This led to the conclusion that the hydrogen bond network and protein environment plays a pivotal role in the redox potential of the complex-cluster.^119^

### Substrate Replacement and Radicals inside the Enzyme with Metal Ions

In continuation of the previous study by Jäger et al. (2017), the Que enzyme was further investigated using molecular dynamics (MD) simulations to simulate the protein while replacing the Mg^2+^ needed to stabilize the radical substrate with Ca^2+^ and Na^+^. The simulations showed that the native Mg^2+^ was able to have longer retention times in Michaelis complex conformation. They then went on to calculate vRSE energies using QM/MM techniques and snapshots of the MD simulation, comparing different levels of the QM/MM protocol of the Que enzyme to the Ca^2+^ and Na^+^. After investigating what is necessary of the metal-substrate complex in the enzyme the study was taken one step further and a protocol for substrate replacement for SAM Que was developed using a mix of substrate docking and RSE values.^79^

### Fine Tuning and De Novo Design for Radical Enzymes

Enhancements to the catalytic activity of enzymes can be made through rational design, and the techniques discussed previously support enhancements and fine-tunings. When the natural enzyme is well-known, promiscuous activity (or activity with a non-native substrate) can be enhanced through structural similarities for other well-known enzymes and mutations of the active site that lower transitions state activation energies and promote activity.^120^ For PFAS degradation, substrate promiscuity is an important parameter because enzymes with broad substrate specificity will have broader applications in the treatment of various classes of PFAS. To date, however, there has been no report that demonstrate that significantly altering substrate specificity by rational design is feasible. Outside of promiscuous capabilities, mutating enzymes still largely relies on structural similarities from the same family of enzymes and on directed evolution.

*De novo* design with the inside-out approach has been reviewed quite a bit within the last ten years, as well as *de novo* development of redox proteins, metalloproteins, and synthetic enzymes in general.^3,121,120,122−127^ The inside-out approach is so named due to starting at the active site and reaction mechanism and working outward. The standard model of the inside-out approach involves creating a theoretical enzyme (theoenzyme). This incorporates the transition state of the desired reaction and a few amino acid residues or their functional groups that assist in reaction. Theoenzymes can also include non-natural amino acids and cofactors. Most likely a variety of theoenzymes will be built for additional the next steps. Once each has been optimized, an algorithm that can either incorporate the substrate or both the substrate and functional groups is used to place the structure into a predefined scaffold. The initial match will then be further optimized at the active site through mutations using automated software such as RosettaDesign. Finally, the design will be evaluated before experimental resources are involved. This can be done through MD simulations or through QM/MM methods to assess the final enzyme catalytic properties. This enzyme can then be further enhanced through directed evolution or MD and QM/MM evaluation and mutation.^3,120^ Completely *de novo* enzymes typically have poor activity rates due to the scaffold enzyme structure which causes failed hydrogen-bond systems, water entering active sites, or steric interactions, along with dynamic motions of the enzyme and changes in the substrate positioning and structure.^120^ The importance of dynamic motion and correlation with activity is discussed in the Head-Gordon and Welborn 2018 review.^120^

### The Inclusion of a Metal Cofactor into a Completely De Novo Enzyme Design Complicates an Already Difficult Process

There have been a number of reviews written on this^122−127^ and it is not within the scope of this review to reiterate all techniques, but some key techniques and examples of recent successes are highlighted here. For example, a number of techniques rely on residue building blocks to coordinate a metal-complex, identifying high affinity of similar scaffold enzymes, and optimizing the second and third coordination spheres. There has been success in designing metal binding sites in α3D structures, a lone polypeptide that is able to fold into a three-helix structure, that then went on to have been successfully expressed and purified,^128^ while another *de novo* designed Zn(II) metalloprotein was able to stabilize semiquinone.^129^ In a third example, a heteronuclear heme-[4Fe-4S] cofactor designed to reduce sulfite with a cytochrome *c* peroxidase scaffold with improvements to the binding sites near the secondary coordination sphere achieved catalytic results close to the native enzyme, sulfite reductase.^130^ The use of enzymes for biocatalyst radical polymerization has been recently reviewed^131,132^ as have metal-protein materials that have applications in catalysis as well as other areas.^133^

Ultimately, when trying to design an enzyme that catalyzes reactions not found in nature there are two underlying issues. One is creating the theoenzyme, due to the reactivity pathway and possible essential residues being unknown. The second is the slower turnover rate compared to a naturally evolved enzyme. While this can be improved somewhat through a combination of laboratory-based directed evolution and optimization, the desired reaction must still be possible.

## Exploring Potential Routes for PFAS Bioremediation

The route for PFAS remediation is a complicated process that may require a variety of interacting parts and is likely to be compound specific. The first part of this review discussed the catalytic redox systems and the inherent need for a reductant/oxidant of at least −1.1 V/2.0 V for PFAS. A high reductant to initiate the reaction, as with aqueous electrons followed by an oxidant, may be the best approach. The inclusion of metal complexes may also help facilitate the reaction by either bringing PFAS closer to the reactant or lowering energy barriers. The weakest bonds within PFAS and their redox routes, the radical stabilization energies and how they may affect the overall degradation route are also key factors. Considering all the tuning and *de novo* techniques discussed above, there are several routes enzymatic remediation could take. The basics of these routes can be adopted and optimized for specific members of the PFAS family, their intermediates and environmental degradation products, and perhaps other hard to degrade pollutants.

### Laccase/Peroxidase

Catalytic reaction mechanisms on heme haloperoxidases have been studied computationally.^134^ The energy barriers of the known pathways are important to understanding how to switch substrates in an enzyme. As discussed previously H_2_O_2_ alone is not capable of breaking the C–F bond, and neither can the heme iron(III)-hydroperoxo. Therefore, this could be used as a secondary enzyme in the degradation route comparably to the hydroxyl radical in the hydrated electron systems. Another potential route may be to follow fine-tuning of metal-complexes with their second-coordination spheres. Studying active-site replacements may help increase the redox potential enough to break the TBA-F bond, which could not be done previously. From here, the substrate might be gradually evaluated for longer chain length PFAS once the C–F bond can be readily broken.

Laccase and peroxidase as PFAS remediation materials have been explored, showing the best remediation route with the use of the HBT substrate with a specific metal ion in solution to bind with the particular PFAS, such as Cu^2+^ for both PFOA and PFOS. The use of specific metal ions to bring PFAS closer to the enzyme and/or lower the activation energy for the radical attack increases the remediation rates. Mechanistic kinetics and understanding may be improved with RSE comparisons for the substrate radical and PFAS radicals created. However, the easiest route for remediation may lay in the combined use of enzymes and materials to serve as sorption sites for PFAS.

An interesting observation was found for a bionanocatalyst (BNC) consisting of HRP and magnetic nanoparticles (MNP). The small MNPs were able to increase turnover rates in the bound enzymes but no change on inhibition was found. The larger MNP clusters were able to not only increase turnover rates but lower inhibition concentration as well, indicating the MNPs were directly responsible for the turnover rate, while the structure was responsible for the lower inhibition concentration. Enzyme inhibition and turnover rates are key influences on remediation abilities, and the use of BNC with MNPs on other radical enzymes could improve them as tools for future use.^135^

### FAcD.

**Figure 8:**
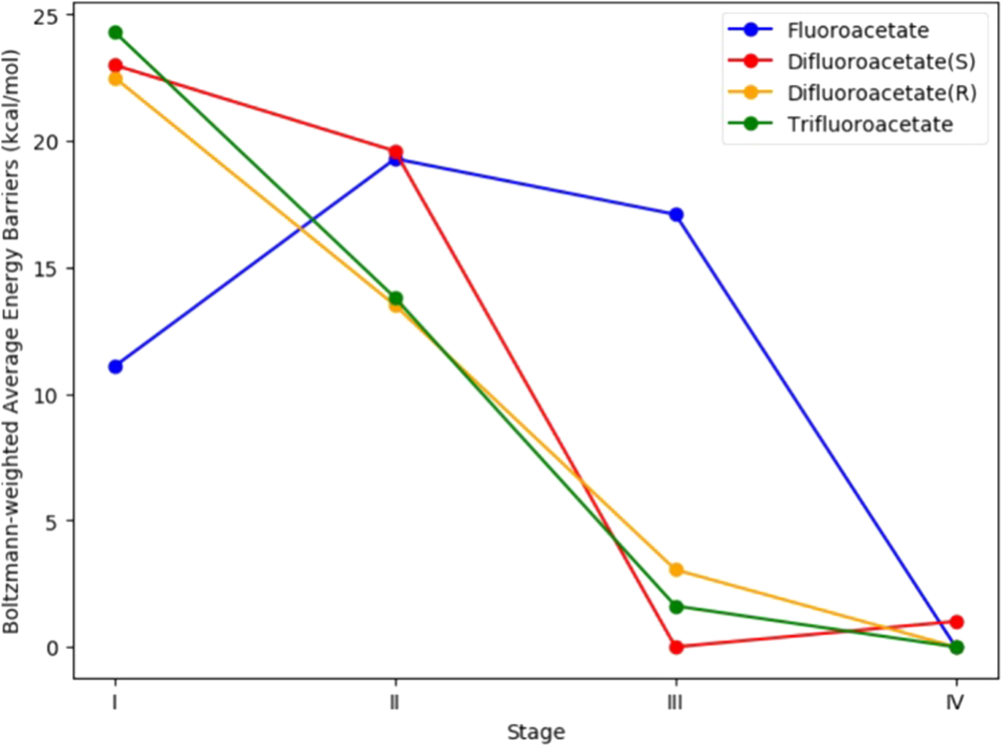
Average energy barriers for 4 stages in the FAcD mechanism for fluoroaceate, difluoroacetate (S and R), and trifluoroacetate. Stage I: C–F activation, stage II: nucleophilic attack, stage III: C–O bond cleavage, and stage IV: proton transfer. These values come from the Supporting Information in Yue Y. et al. 2021.^94^

The next step in further developing this enzyme would be expanding upon its ability to defluorinate a tertiary substituted carbon, increasing the chain length of substrates that could be accommodated, or the use of fluoromethanesulfonate as a substrate (being closer in structure to sulfonate PFAS). A possible route for further substituted remediation is to follow EVB to screen the active site of various ensembles generated from MD, paying special attention to the halogen pocket and any other electron-transferring amino acids and key residues. Transition states of the S_N_2 attack and nucleophilic attack would be most useful. However, the optimization of this stage would have to be evaluated against the rest. In Figure 8 the Boltzmann-weighted average energy barriers as calculated by Yue et al.^94^ are shown. As we optimize for stage I we would have to evaluate the effect on the other three stages. These mutations can be further run through MD to evaluate the dynamics of the mutated enzyme before experimental evaluation.

### SAM/B12

Since oxidizing agents may break C–F bonds, C–C bonds, or remove functional groups, and the SAM mechanism works through creating an AdoMet radical which quenches its radical via a hydrogen abstraction, PFAS that contain hydrogens and have the potential for rearrangement would be best fits for these enzymes. Another interesting part of the active site of this enzyme is the Mg ion that helps balance the RSE. PFOS was shown to complex with Mg^2+^, which may help with radical stabilization and/or reduction of sulfonated headgroup PFAS. A protocol set forth by Jäger et al. 2019 uses mass docking of substrates, RSE, and vRSE MD to screen possible substrate alternatives for the SAM enzyme.^79^ The RSE values can be compared against those calculated for active substrates and further optimized through active site mutations. The general sense of RSE for PFAS could be applied here to help with the RSE screening process. This protocol can also be applied to other reactions substituting the RSE with another thermodynamic profile that follows Hammond’s postulate in which the reaction intermediate is closely related to the catalytic rate.^79^

The B12 dehalogenators PCeA and NpRdhA use their cob(II/I)alamin for homolytic cleavage of the C–Cl bond. Reduction potential studies using density functional theory (DFT) have studied the mechanism for dechlorination of PCE, TCE, and MCE. These studies used redox potentials and energy barriers of the elementary mechanism and transition states to evaluate the mechanistic pathway of PCE and evaluate the potential for MCE.^87^ The dehalogenation mechanism is carried out by the B12 complex. Gas phase complex studies may be helpful here as a first protocol to determine if defluorination has a better probability of happening as described by the homolytic bond cleavage or the electron transfer or if B12 should be tuned. The redox potential of B12 may be greatly different outside of enzymatic environment with its substrate as indicated in Table 2, with chlorophenol’s reductive potential versus B12 and their respective ability to react. Therefore, some key enzymatic residues should be selected for energy calculations. Docking and MD simulation may also be an effective way to predict the most probable mechanistic route quickly and the best starting PFAS. The distance from the B12 to the substrate may aid in discerning two possible dehalogenation routes discussed (Figure 5) without QM simulations. Once initial determinations in dehalogenation routes are made, from here the protocol would be similar to optimizing the metal-complex and active site to facilitate dehalogenation.

### Theoenzyme

A completely *de novo* design, though challenging, may have potential by drawing on knowledge from experiments and patterns seen in existing dehalogenating enzymes. Many enzymes use metal ions to stabilize their substrates or radicals. Out of the metal ions Fe^3+^, Cu^2+^, Mg^2+^, and Mn^2+^, PFOA has been shown computationally to complex with Fe^3+^ and Cu^2+^, while PFOS was shown to prefer Mg^2+^ and Cu^2+^ based on HBT radical studies. There is also the use of amino acids that can transfer electrons to facilitate mechanistic pathways. The halogenated pocket and nucleophilic Asp residue might be a good place to start, by manipulating a theoenzyme with different supporting residues and metals until an optimal defluorination transition state energy is achieved. Many theoenzymes would have to be generated until an optimal structure is reached, following protocols of previously designed theoenzymes. B12 or a iron–sulfur complex may also be a good starting point for a theoenzyme. This route would focus on enabling reduction or oxidation of PFAS through different metal complexes and supporting second spheres.

Nanozymes, which are nanomaterials with enzyme-like properties, have recently gained attention as promising artificial enzymes because of their potential to have tunable catalytic efficiency, higher stability relative to natural enzymes, and low cost due to their recyclability. While natural laccases have been recognized as versatile biocatalysts, their applications in environmental remediation have been hampered by their intrinsic fragility leading to denaturation and lost activity. Hence, artificial laccases based on nanomaterials containing copper or cerium as active sites are being developed. Nanozymes based on metal organic frameworks (MOFs) with laccase-mimicking activities are promising bioinspired catalysts (Liang et al, 2022) that have been shown to effectively degrade bisphenol A, a persistent environmental pollutant.^136^ Multivalent Ce-MOFs with intrinsic redox reactivity of Ce^4+^/Ce^3+^ within their active sites mimic the redox Cu^2+^/Cu^+^ electron transfer pathway of natural laccase showing great potential for environmental remediation. The potential applications of MOFs-based nanozymes for PFAS degradation have not been explored, although MOFs have been used to sequester PFAS from aqueous solutions for potential remediation.^137,138^

## Conclusions and Future Outlook

The breaking of carbon–fluorine bonds is no easy task. Most current technologies discussed in this review involve saturating solutions with high-energy radicals. A better understanding of the intermediate steps of this process is necessary in order to control the reaction and optimize it for remediation treatments. Experimental and computational studies should be combined to obtain a better understanding of thermodynamic pathways. The ability of PFAS to form complexes with metal catalysts also needs further study. Their potential to lower activation energy or increase proximity for radical attack would be of great use in designing PFAS remediation systems. Bacteria that can actively defluorinated PFAS have yet to be mapped out and the enzyme(s) responsible discovered. Without this information, and with so few defluorination enzymes known, enzymatic degradation for the near future may benefit from knowledge gained through molecular simulations and both *in silico* and *in vitro* interactions with various metal-complexes and co/substrates. The goal of molecular modeling need not focus on designing an extensive library of mutants, but to narrow down the list of mutants to a few so that they can be experimentally assayed for activity and specificity. To achieve this goal, it is critical to efficiently combine molecular modeling and experimental validation very early in the design process in order to achieve a good balance between substrate promiscuity and conversion rate. In this regard, monitoring of reaction intermediates and their kinetics of formation will be key in the optimization of the catalytic efficiencies of engineered enzymes for PFAS degradation. A combination of sensitive analytical techniques such as high-resolution LC/MS-MS and specific characterization instruments such as ^19^F nuclear magnetic resonance (^19^F NMR) can be used in the identification and quantification of unknown intermediates and byproducts, which are necessary information needed for designing efficient enzymes in PFAS remediation. Finally, since HRP and laccases have both been demonstrated to catalyze degradation of PFAS, and that nanozymes have been shown to have peroxidase-like or laccase-like activities, it would be worthy to explore the potential of MOFs-based nanozymes for the capture and destruction of PFAS from environmental samples.

## Data Availability

As this is a review paper, we have no data or software generated in this study to report.
